# Perceptions of In-home Monitoring Technology for Activities of Daily Living: Semistructured Interview Study With Community-Dwelling Older Adults

**DOI:** 10.2196/33714

**Published:** 2022-05-05

**Authors:** Nicola Camp, Julie Johnston, Martin G C Lewis, Massimiliano Zecca, Alessandro Di Nuovo, Kirsty Hunter, Daniele Magistro

**Affiliations:** 1 School of Science and Technology Nottingham Trent University Nottingham United Kingdom; 2 Kurio 3D Compression Ltd Mansfield United Kingdom; 3 Wolfson School of Mechanical, Electrical and Manufacturing Engineering Loughborough University Loughborough United Kingdom; 4 Institute of Electrical and Electronics Engineers Sheffield Hallam University Sheffield United Kingdom

**Keywords:** aging, wearable sensors, environmental sensors, social robots, activities of daily living, aging, older adults, elderly, robots, wearables

## Abstract

**Background:**

Many older adults prefer to remain in their own homes for as long as possible. However, there are still questions surrounding how best to ensure that an individual can cope with autonomous living. Technological monitoring systems are an attractive solution; however, there is disagreement regarding activities of daily living (ADL) and the optimal technologies that should be used to monitor them.

**Objective:**

This study aimed to understand older adults’ perceptions of important ADL and the types of technologies they would be willing to use within their own homes.

**Methods:**

Semistructured interviews were conducted on the web with 32 UK adults, divided equally into a *younger* group (aged 55-69 years) and an *older* group (≥70 years).

**Results:**

Both groups agreed that ADL related to personal hygiene and feeding were the most important and highlighted the value of socializing. The *older* group considered several activities to be more important than their younger counterparts, including stair use and foot care. The older group had less existing knowledge of monitoring technology but was more willing to accept wearable sensors than the younger group. The younger group preferred sensors placed within the home but highlighted that they would not have them until they felt that daily life was becoming a struggle.

**Conclusions:**

Overall, technological monitoring systems were perceived as an acceptable method for monitoring ADL. However, developers and carers must be aware that individuals may express differences in their willingness to engage with certain types of technology depending on their age and circumstances.

## Introduction

### Background

The global population of people aged ≥60 years is projected to increase to >2 billion by 2050 [1]. In the United Kingdom alone, one-quarter of the population is expected to be aged ≥65 years by 2050 [2]. Although modern medical care has facilitated this rise in life expectancy, it has also increased the length of time that individuals are likely to require long-term care [3]. Often, the systems in place to provide this care are poorly equipped to do so in an effective manner [4]. A potential solution to this may be to create smart environments that support older adults’ ability to live independently in their own homes, which would reduce the need for care home facilities and allow them to focus on those with the most severe difficulties [5]. Moreover, many older adults prefer to remain in their own homes for as long as possible [6]. To determine their suitability for home care, if needed, an individual is assessed on their activities of daily living (ADL) performance [7].

ADL are any of the activities that are fundamental for an individual to live independently [8]; for example, feeding, washing, and mobility. Several scales and methods can be used to assess ADL function, including a variety of activities ranging from the very basic to more complex activities (instrumental ADL [iADL]) [9]. One of the key scales that is often used because of its inclusion of both basic ADL and iADL is the Groningen Activity Restriction Scale (GARS) [10]. However, it has been proposed that these scales may lack accuracy and objectivity; therefore, technological solutions have been proposed as alternative methods for assessing ADL [9]. It has been suggested that encouraging the use of monitoring technology may help to maintain the levels of autonomy [11], allow older adults to acknowledge their own needs in terms of assistance [3], and help caregivers to provide interventions or assistance at the most appropriate time or at a suitable level for the individual [12].

Despite their potential, many older adults are unaware of the existence of monitoring technology; therefore, they are seldom used [11]. Monitoring technologies can typically be divided into 2 broad categories.

- Wearable sensors are sensors with some physical attachment to a person, such as a wrist-worn device [9].
- Environmental sensors are sensors placed around the home with which a person does not necessarily need to have any direct interaction but which will monitor activity within a room, such as a motion sensor [9].

Of those who are aware of the technology, there is often a reluctance to embrace it, which may be because of diminished openness to new experiences or feelings that the technology may be too advanced for their abilities [13]. However, few studies have focused on older adults’ perceptions of the monitoring technology; therefore, the reasons for their limited use remain unclear. A comprehensive study of technology aimed at assisting older adults in their own homes, which included some monitoring technology [11], found that although technology may offer some solutions, it is not yet well-integrated into the daily care of older adults and is less accepted, especially among older adults (aged ≥65 years). These findings are echoed by Berridge and Fox [14], who found that adult children were more willing to use technology than their older parents, although older adults were able to comprehend their use. A review of fall-monitoring technology [13] noted that older adults approach technology differently than their younger counterparts but are showing increasing rates of adoption. Therefore, continually questioning the utility and acceptance of new technologies remains relevant [11].

In the studies by both Berridge and Wetle [14] and Verloo et al [11], the older adults were already in need of some form of home care, which implies that they were in a state of decline. The aim of many specific ADL monitoring systems is to identify individuals before they reach this stage [9]. Therefore, there is a need to understand the perceptions of both *younger* older adults—those who are less likely to require assistance at the time of installation—and *older* older adults who may already be experiencing some form of physical or cognitive decline. It is anticipated that by engaging with older adults and understanding their perspectives on the activities that they consider important to live, as well as their opinions on the types of technologies that can monitor them, future developments in this area will be better accepted by the older adults who they aim to help.

### Objectives

This study aimed to understand the perceptions of both *younger* older adults (aged 55-69 years) and *older* older adults (≥70 years) related to ADL monitoring technology and the activities that they should monitor.

### Theoretical Framework

This study was guided by the theoretical framework developed by Peek et al [15] and subsequently used by Verloo et al [11]. This framework provides us with some basic foundational components that have been shown to influence community-dwelling older adults’ acceptance of technology, including perceived concerns, perceived benefits, and older adult characteristics.

## Methods

### Design

This study used a qualitative design to collect data on the perceptions of older adults using one-to-one and photo-elicitation interviews (PEIs). Reporting on the study was based on a checklist for explicit and comprehensive reporting of qualitative studies [16].

### Population and Settings

This study included community-dwelling older adults aged ≥55 years. All participants lived in the United Kingdom without a medical prescription for home care. All interviews were conducted on the web using the video call software Microsoft Teams (1/33, 3%), Zoom videoconferencing (24/33, 73%), WhatsApp (6/33, 18%), and Facebook Messenger (2/33, 6%).

### Participant Recruitment

Participants were recruited through social media and email contact from charity groups, including Age UK and the University of the Third Age. To be included, participants had to be aged ≥55 years, be able to live independently in the community without receiving specific home care, and have access to a form of video call software. Participants were divided into 2 groups: *younger* (aged 55-69 years) and *older* (aged ≥70 years).

### Ethics Approval

This study was approved by the institutional human research ethics committee (18/19–75V2).

### Data Collection Procedure

Data were collected between July 21, 2020, and February 2, 2021. Older adults who expressed interest in participating via social media platforms and through email contact with older adult charity groups, including Age UK and the University of the Third Age, were contacted and provided with written details of the study. Once they were given an opportunity to reflect on the study requirements, a date, time, and video call software were agreed upon. On the day of the interview, the interviewer verified that participants understood the information that had been provided to them and gained verbal consent that they were happy to continue with the interview. Data collection used 14 photographs of relevant technologies and a semistructured interview guide (Multimedia Appendix 1). Interview audio was recorded for subsequent transcription.

### Data Collection Instruments

#### Overview

The research team developed and tested semistructured interview guides and PEIs (Multimedia Appendix 1). These guides used open-ended questions to encourage participants to discuss their thoughts on ADL and monitoring technologies (wearable and environmental-based systems). The interviewer had the freedom to reformulate, reorganize, or clarify questions during the interviews to gain a deeper understanding of the community-dwelling older adults’ thoughts and opinions. The guidelines were divided into 2 broad categories: ADL and monitoring technologies.

#### ADL Instrument

Participants were asked, “What activities do you consider fundamental to your daily life?” and then showed the activities included in the GARS. They were asked to rank these activities on a scale of 1 (low importance) to 5 (high importance) and encouraged to explain their decisions. Following this, they were asked whether there were any activities they felt were important but not included in the GARS and asked to describe what they thought would make these ADL difficult to perform in the future.

#### Monitoring Technology Instrument

Information was collected primarily using PEIs, where participants were shown image examples of wearable and environmental sensors during their interviews (Multimedia Appendix 1). They were asked whether they had any awareness of each technology type, and then, the purpose of each was explained. Participants were asked the following: “What do you like/dislike about the technology shown here?”; “What do you think the benefits of using this technology to monitor activities of daily living might be?”; and “What concerns do you have with the use of these types of technology?”

### Data Analysis

We recorded 18 hours and 32 minutes of interviews and PEIs (mean 42, SD 12 minutes). All interviews were transcribed verbatim from the audio recordings. The data were analyzed using a realist thematic analysis approach [17]. One of the key advantages of this approach is the appreciation of both quantitative and qualitative data, which can be gathered from interviews [18,19]. A total of 3 authors (NC, DM, and JJ) were involved in the analysis of the transcripts, with a collective discussion to finalize the included codes.

The first interview transcription was analyzed, and initial codes were identified, which were then grouped and refined into themes. Using a deductive approach, the second interview was analyzed, similar themes were identified, and additional themes were added. This process was continued for each interview transcript, each time adding or refining the existing themes. By adopting this data-driven approach, it was possible to continuously test the truth of emerging themes, allowing some quantitative aspects of this research to be obtained simply. The realist thematic approach allows quantitative-type information to be collected, such as the frequency of a theme (indicating strength) and the number of participants expressing similar thoughts or experiences (indicating prevalence) [17]. After all the interviews were analyzed, the list of themes was checked and compared with another investigator to identify any disparities between them. If any disputes arose, the original transcript was checked, and the dispute was settled through discussion between the researchers. The data were stored and analyzed using Microsoft Excel (Multimedia Appendix 2).

## Results

### Samples and Sociodemographic Data

This study included 33 community-dwelling older adults from the United Kingdom; 17 (52%) *younger* and 16 (48%) *older*. The younger group comprised 17 community-dwelling older adults (n=9, 53%, women and n=8, 47%, men) aged 55 to 67 (mean 61.9, SD 4.0) years. The older group comprised 16 community-dwelling older adults (n=9, 56%, women and n=7, 44%, men) aged 70 to 81 (mean 74.0, SD 4.5) years.

### Findings

#### Overview

We have divided the description of our results into two main sections: *ADL Findings* and *Monitoring Technology Findings*. *ADL Findings* include (1) factors that influence the perceived importance of GARS activities, (2) additional activities, (3) factors that may influence ADL performance, and (4) factors that influence the acceptance or rejection of assistance in performing ADLs. *Monitoring Technology Findings* is divided into three subcategories: (1) general monitoring systems, (2) wearable sensors, and (3) environmental sensors. Within each of these, the results are further divided into factors influencing potential acceptance, potential advantages, and potential disadvantages. Participants did not highlight any general disadvantages but made some suggestions for future development, which are given at the end of this section. An example quote for each identified element is provided, along with an indication of how many participants shared the same sentiment. More examples can be found in the accompanying Microsoft Excel file (Multimedia Appendix 2). The sample size included in this study was not large enough to accurately provide statistical differences between groups. However, the realist approach to the adopted analysis allows quantitative data to indicate the strength and prevalence of participants expressing similar thoughts or opinions [17].

#### ADL Findings

##### Factors That Influence Perceived Importance of GARS Activities

**Figure 1:**
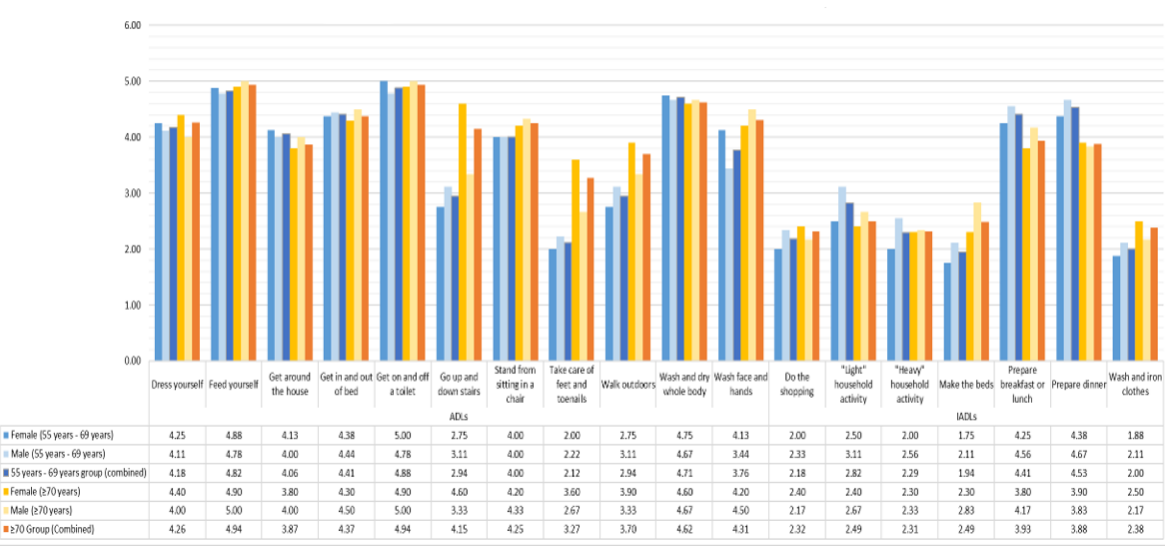
Relative importance of Groningen Activity Restriction Scale activities.

The perceived importance of the GARS activities is summarized in Figure 1. In general, ADL received higher importance overall than iADL, except for iADL related to food. Both the younger and older groups ranked *get on and off toilet*, *feed yourself*, and *wash and dry whole body* as the most important ADL. The ≥70 years group placed more importance on *go up and down stairs*, *take care of feet and toenails*, and *walk outdoors* than the 55 to 69 years group, especially women. The 55 to 69 years group placed more importance on *light household activity*, *prepare breakfast or lunch*, and *prepare dinner* than the ≥70 years group. *Make the beds*, *do the shopping*, and *wash and iron clothes* were considered of the lowest importance, especially in the 55 to 69 years group. The statements provide some greater context relating to why activities were deemed *high importance* or *low importance*.

In general, of the 33 participants, the activities considered *high importance* related to maintaining physical function, as described by 10 (30%; younger woman, n=1, 10%; younger men, n=2, 20%; older women, n=5, 50%; and older men, n=2, 20%) participants:

If you can keep yourself active then, through things like getting in and out of bed and, you know...or on the toilet, or getting off the toilet, then it all comes, you know, under that one umbrella, so to speak, of keeping yourself active

027BB

Alternatively, the activities considered of *high importance* were because of existing conditions, explained by participant 010BC (younger man, 1/30, 3%) as follows:

I’m diabetic, I have to keep an eye on it [taking care of feet and toenails]—you know, your feet are quite important

010BC

Of the 33 participants, many participants viewed the relationships between activities as an important factor, such as getting in and out of bed for 5 (15%; younger man, n=1, 20%; older women, n=2, 40%; and older men, n=2, 40%) participants, getting in and out of a chair for 5 (15%; younger men, n=3, 60%; older women, n=1, 20%; and older man, n=1, 20%) participants, moving around the house for 1 (3%; older woman) participant, taking care of feet and toenails for 4 (12%; older women, n=3, 75%, and older man, n=1, 25%) participants, light household activity for 1 (3%; younger man) participant, and shopping for 2 (6%; younger woman, n=1, 50%, and older man, n=1, 50%) participants:

well getting out of bed is, you’ve got to do that to do everything else

029MG

I mean standing up from sitting in a chair, again, you’re not going to be very independent if you can’t do that

021GD

you have to do everything—you need to be able to [move around the house] to do everything else

006BR

if you’ve got problems with your feet you won’t be able to [do] much of the other stuff

013MB

it’s all about having clean crockery for use in the kitchen—you can’t cook for yourself without clean crockery so that goes with preparing food and feeding yourself...I mean I could eat off a dirty plate if I couldn’t wash up, it’s just not healthy

032TB

it’s still important [shopping] because I think you need to get out and socialise as well don’t you, the older you’re getting.

012SH

Of the 33 participants, the ability to perform the shown ADL was linked to the idea of maintaining pride and dignity, especially among the younger group, as mentioned by 12 (36%; younger women, n=4, 33%; younger men, n=4, 33%; and older men, n=4, 33%) participants:

it’s anything that takes that confidence away, and your self-esteem, it just rips it apart...it’s just demoralising, [performing ADLs] is vitally important because if you are dirty, or smelly, you just don’t feel nice about yourself.

024SK

Of the 33 participants, walking outdoors was considered an important activity because of its relationship with mental health in both the younger and older groups, as described by 7 (21%; younger woman, n=1, 16%; younger men, n=4, 67%; older woman, n=1, 17%; and older man, n=1, 17%) participants:

“walk outdoors” I think is essential for mental health, but it’s not absolutely necessary...I think it’s essential for mental health but, er, not for existing

019GW

Of the 33 participants, regarding low-importance activities, the acceptance of assistance, either mechanical or human, was a key factor, as described by 12 (36%; younger women, n=5, 42%; younger men, n=4, 33%; older men, n=3, 33%) participants:

With things like the household activity and the ironing, if I got to the stage where I couldn’t do that I would pay somebody to do it, so I don’t regard that as a heavy priority because—the same as do[ing] the shopping, I mean we’ve been having food, um Tesco, deliveries so I don’t regard them as a big thing because you can get somebody else to do it couldn’t you. Same as make the beds

007JR

For some of the 33 participants, some activities were less important as they were considered autonomous, as described by 4 (12%; younger men, n=2, 50%, and older women, n=2, 50%) participants:

and of course, getting in and out of bed and making beds, well you just do these things automatically without even thinking about it

018CW

Of the 33 participants, some activities were considered less important as they had little impact on everyday function, as described by 8 (24%; younger woman, n=1, 13%; younger men, n=3, 37%; older women, n=3, 37%; older men, n=1, 13%) participants:

One can always live in a house that is not that tidy and not, um, it’s not going to affect whether you are, sort of, capable of fending for yourself. If the house gets dirtier then it’s not the end of the world

021GD

However, several of the 33 participants noted that the specificity of the activity being considered would have an impact on its difficulty and importance; for instance, making versus changing the beds for 4 (12%; younger women, n=2, 50%; older woman, n=1, 25%; and older man, n=1, 25%) participants, meal preparation for 6 (18%; younger woman, n=1, 17%; younger man, n=1, 17%; older women, n=3, 50%; and older man, n=1, 17%) participants, and household activity for 12 (36%; younger women, n=3, 25%; younger men, n=4, 33%; older women, n=3, 25%; and older men, n=2, 17%) participants:

“making the beds” I think depends on how much making the beds—if you are just pulling it straight it’s fine but if you are going to re-cover a duvet after its been washed, that’s probably a bit too heavy for a lot of people

019GW

I mean there is slightly, but I would put them together for the purposes of this, yeah, well, because to my mind you need more motor skills to prepare dinner than you do for a breakfast or a lunch...but it needn’t be [more complex] because you can always prick holes in something and stick it in the microwave

020PP

changing the bed, or turning a bed, hoovering which involves pushing, that’s quite physical, um, and maybe getting the washing and hanging the washing out maybe. Or, you know, bending down to get it out of the washing machine, that’s quite—I would call that heavy. But light stuff, like maybe putting the duster round or, um, like you said, a little bit of washing up, not too much of a problem

024SK

##### Additional Activities

**Figure 2:**
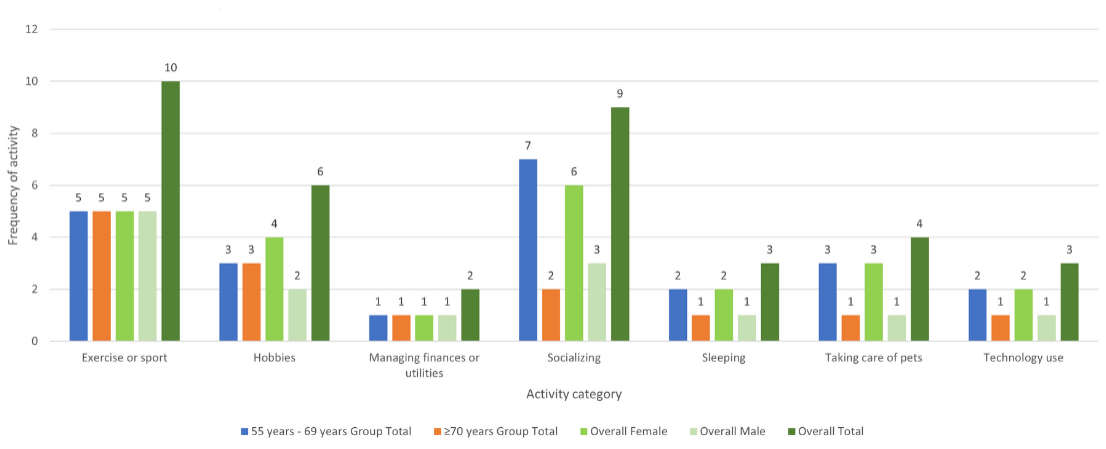
Frequency of additional activities. Exercise or sport refers to moderate to vigorous physical activity such as exercise classes but not walking outdoors, as this is specified in the Groningen Activity Restriction Scale. Hobbies refer to low-intensity activities, often with a social aspect, such as crafts, poetry groups, and choirs.

During the interviews, the participants identified 7 extra activities that were not present on the GARS (Figure 2). Exercise or sports (10/33, 30%) and socializing (9/33, 27%) were the most frequent additional activities.

Exercise or sport was cited most frequently, explained by participant 008AE (older woman, 1/33, 33%) as follows:

I’ve always done yoga so I do believe that you have to use your body rather than sit in a chair and go arthritic. So I think I look at it and my attitude is a little bit different by doing yoga for a lot of years and knowing I got to keep my body mobile

008AE

Socializing was cited most frequently by those aged 55 to 69 years, with mental health being a commonly cited reason for its importance, explained by participant 022CB (younger woman, 1/33, 33%) as follows:

I think that is important because if you’ve got, see, people socially—*sigh*—there’s nothing worse than being alone, because you get depressed...I think that ought to be mentioned, meeting people and socialising with people

022CB

##### Factors That May Influence ADL Performance

Of the 33 participants, one of the main factors that community-dwelling older adults consider to influence their ADL performance, either currently or they perceive will influence performance in the future, is their housing situation, as described by 13 (39%; younger women, n=4, 31%; younger men, n=3, 23%; older women, n=4, 31%; and older men, n=2, 15%) participants. Interestingly, for the women among the 33 participants, this was because of who they lived with and how they divided ADL between them, as described by 7 (21%; younger women, n=2, 29%; younger men, n=2, 29%; and older women, n=3, 43%) participants:

I’m sort of in charge in the kitchen I suppose...I cook, and he said he washes up but he means “loads the dishwasher” and he does generally do the hoover—he does generally get the hoover out, so yeah, I suppose we do things between us really, yeah.

028PG

In contrast, the men among the 33 participants tended to focus more on the practical environment, as described by 5 (15%; younger women, n=2, 40%; younger men, n=1, 20%; and older men, n=2, 40%) participants:

depending on where you live you’ve got to get up and down the stairs

019GW

Of the 33 participants, the most common influencing factor in ADL performance was physical ability, as described by 13 (39%; younger women, n=4, 31%; younger men, n=3, 23%; older women, n=4, 31%; and older men, n=2, 15%) participants:

it’s just as your body gets weaker and your joints start to pack up, erm, I mean a lot of those—anything that requires real physical movement, they’re the ones that can get difficult when you get so much older.

025JD

Mental health was cited as an important factor by one of the participants (younger women, 1/33, 3%*)*:

because if you’re depressed you don’t feel like getting out of bed, but if you physically can’t get out of bed, that’s frustrating and, um, also it might make you feel depressed because you can’t get out of bed

024SK

In relation to exercise, self-control was highlighted by one of the participants (younger man, 1/33, 3%):

I know I should be doing exercise and I know I shouldn’t be eating fatty foods so, you know, it’s down to me if I choose to do it or not and then it’s down to me what the consequences are. I know the consequences, I know the rules so, you know, it’s down to me and I should really just stick with it

017SC

##### Factors That Influence the Acceptance or Rejection of Assistance in Performing ADL

Of the 33 participants, maintenance of pride or dignity was a key factor in community-dwelling older adults resisting assistance with ADL, as well as the embarrassment of having to rely on someone else, as described by 4 (12%; younger woman, n=1, 25%; younger man, n=1, 25%; and older women, n=2, 50%) participants:

imagine having a complete stranger come in and have to work with you quite intimate—well, very intimate—I mean, it’s just not natural, you know...You know, if you have a completely new carer come in to [do] these things for you, you haven’t instantly got a rapport. I mean, you feel embarrassed...there’s embarrassment because human beings do not naturally, you know, expose private actions to complete strangers

024SK

Cost was another factor, especially in relation to mechanical assistive technology such as stairlifts (younger woman, 1/33, 3%):

If you can pay for your own Stanna lift then fantastic and it wouldn’t necessarily be a problem but if finance is a problem then going up and down stairs might be

024SK

In contrast, others stated that they would be willing to accept assistance if they physically needed it or if it would improve their ability to remain independent (older women, 2/33, 6%):

I’ve got a seat in my shower that I never use. I had that fitted—I did have that. I did say “can you fit me a seat, for when I need it,” and I did—I think I used it after I had my hip done, because I thought “better be on the safe side—I’ll sit on the seat.”

022CB

#### Monitoring Technology Findings

##### Overview of Monitoring Systems in General

**Figure 3:**
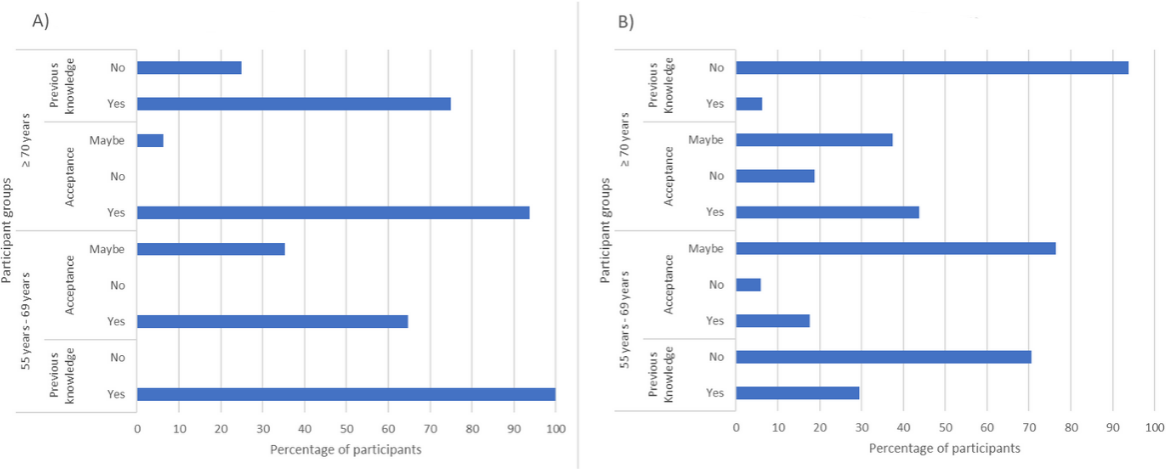
Summary of existing knowledge and overall acceptance of types of monitoring technology; (A) wearable sensors and (B) environmental sensors.

The existing knowledge and acceptance of wearable sensors and environmental sensors are summarized in Figure 3. Overall, the younger group had more existing knowledge of monitoring technology than their older counterparts did. When asked if they would use the technology, the older group was more likely to accept it without delay, whereas the younger group was more likely to say that they would consider using it in the future.

##### Factors Influencing Potential Acceptance of Monitoring Systems in General

Of the 33 participants, for both groups, health status and general technology acceptance were the key influencing factors for 10 (30%; younger women, n=2, 20%; younger men, n=2, 20%; older women, n=4, 20%; and older men, n=2, 20%) participants:

I do think that when you live alone, whatever your stage of mobility, you could fall over at any old time can’t you, so...yes. A reserved yes [to having some kind of system]...because I don’t like to think that I’m quite at the stage where I need it yet. But that the whole point, like, you should have them before you need them

020PP

I do try but I find it difficult and I think you do as you get older but I do try [using a] mobile phone and I’m trying to use the iPad. I don’t say I find it easy but I have to keep trying because I think you have to learn to do these things because that’s the way of the future isn’t it? To have to use these gadgets

008AE

The younger age group mentioned experience with technology as an influencing factor when considering monitoring technology (younger women, 4/33, 12%):

I think a lot of it is confidence, and so many people I know who can’t manage with like the portal, and the internet and all the different ways—it’s because they don’t have enough expertise in it. We were born in a generation where [there was] nothing like that

016AA

However, of the 33 participants, it was noted that this might become less of an issue in the future by 8 (24%; younger women, n=3, 23%; younger men, n=2, 15%; older women, n=2, 15%; and older men, n=1, 8%) participants:

I think it’s the generations are getting older and they’re not so worried about technology. It’s just like a day-to-day thing for us but when you’re sort of in your 80s now you’ve never been used to it.”

003MC

In contrast to these positive influences, of the 33 participants, 4 (12%; younger women, n=1, 25%, and younger men, n=3, 75%) participants from the younger group suggested that monitoring technology is limited in its usefulness; therefore, they would be hesitant to use anything:

I have a bit of scepticism here because if somebody thinks they are being monitored, I mean theres the sort of “whats in it for them” and if they feel that people are just checking up on them, erm again thinking to my Mum, she would be quite canny so a device could easily be fooled.

009JJ

Of the 33 participants, the issue of data use was also highlighted by 2 (6%; younger woman, n=1, 50%, and older man, n=1, 50%) participants:

not specifically about the wearing of it, um, I think there are concerns about the whole data collection issue, and what happens with it, and how secure it is. Um, but I mean the actual, the technology I don’t have a problem with. The problem lies with what people do with the information once they’ve got it

013MB

##### Perceived Advantages of Monitoring Systems in General

Of the 33 participants, the main advantage of monitoring systems was reassurance, especially for the younger group, who frequently considered using them from the perspective of a carer, as described by 5 (15%; younger woman, n=1, 20%; younger men, n=3, 60%; and older woman, n=1, 20%) participants:

Even if not necessarily for you, for your carer or family...they would be able to see if you’re moving around and, I don’t know if the timescale would be on it, but they would know what time you’re moving about

030DG

Of the 33 participants, health monitoring was another advantage, especially the potential use for health care workers or as a means of supporting medical care, as described by 3 (9%; younger woman, n=1, 33%; younger man, n=1, 33%; and older man, n=1, 33%) participants:

I can see that it has got its place, and from a medical point of view, if it’s being fed into a database and it could highlight problems, erm, then that could be good. If it would highlight problems and then a doctor or a medical person of some kind was alerted that you should go and talk to that person, I could see that would be useful.

017SC

Of the 33 participants, the ability to check whether someone was physically active rather than sedentary was highlighted as an advantage by the younger group, as described by 3 (9%; younger women, n=2, 67%, and younger man, n=1, 33%) participants:

as you get worse as you get older, you know, you might lose some sight or something like that, you know, so having a sensor for getting up and down and that sort of thing, they would know wouldn’t they, what, how much they’re moving. I don’t know, yeah. I don’t think it’s a bad idea as you get older

031SG

In addition to monitoring activity, of the 33 participants, 3 (9%; younger women, n=2, 67%, and older women, n=1, 33%) participants suggested that monitoring technology could provide reminders to conduct certain activities:

Obviously as you get older and the old brain cells are going “oh did I go for my walk today,” “oh no I haven’t” so yeah. And maybe I am sitting around more one day than another, so yeah, yeah. Yeah I think they could probably be quite a good tool actually, yeah.

012SH

##### Overview of Wearable Sensors

Participants from both groups stated that they had more knowledge of wearable sensors than other types of technology, with 100% (17/17) of the younger group and 75% (12/16) of the older group expressing existing knowledge (Figure 3). Overall, 100% (16/16) of the older group would accept at least one form of a wearable monitoring system, 94% (15/16) would consider using it immediately, and 6% (1/16) would consider using it in the future. Approximately 100% (17/17) of the younger group would also accept at least one form of wearable monitoring system; however, only 65% (11/17) would consider using it immediately, whereas 35% (6/17) would consider it for future use.

**Figure 4:**
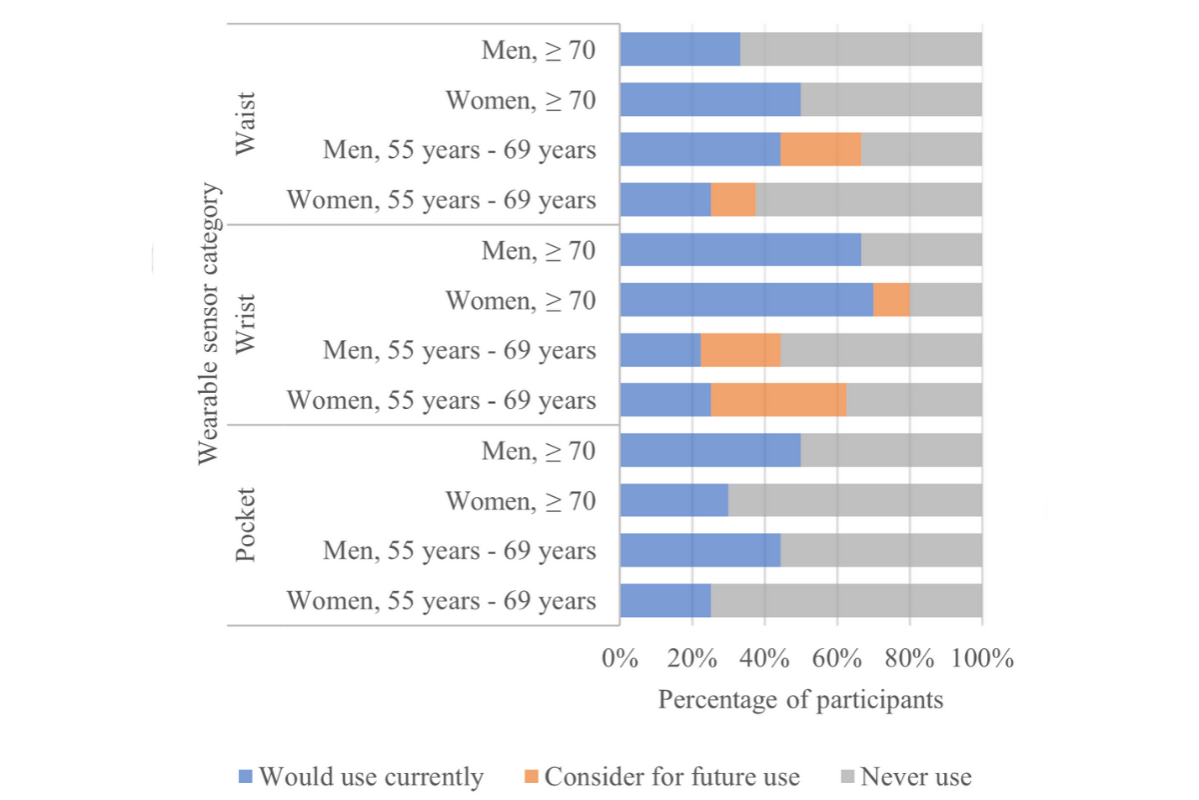
Acceptance of wearable sensors among community-dwelling older adults.

The acceptance of each specific wearable technology type is summarized in Figure 4. Of the 33 participants, wrist sensors were the most acceptable form of wearable technology in both groups, which may reflect the type of technology the participants were accustomed to, as described by 8 (39%; younger women, n=2, 15%; younger men, n=2, 15%; older women, n=3, 23%; and older man, n=1, 8%) participants:

yeah, got them. Got Fitbits. But I know there are all sorts of heart monitors and stuff like that you can wear nowadays

023DK

Although considered acceptable, many of the younger group participants stated that they would not use the wrist sensor currently but would consider it for future use (Figure 4). The same can be applied to a waist-worn sensor, which was the second most acceptable form of wearable technology; however, again, several younger participants would consider it for future use rather than use it immediately. The ring was the least acceptable technology type, although it was slightly more popular among the older group.

##### Factors Influencing Potential Acceptance of Wearable Monitoring Systems

Of the 33 participants, one of the main reasons for the high acceptability rate of wearable technology was how commonly the technology was currently used and how easily these sensors could be combined with other technologies such as a watches, as described by 4 (12%; younger man, n=1, 25%; older woman, n=1, 25%; and older men, n=2, 50%) participants:

well I think people are used to seeing things on people...and it’s not remarkable anymore. I mean I think technology is so widely accepted now that people don’t even comment. Fitbits, you know, people used to say “oh what’s that” but now it’s just a watch.

015AA

However, of the 33 participants, it was suggested by 3 (39%; younger man, n=1, 33%; older woman, n=1, 33%; and older man, n=1, 33%) participants that a wrist-worn sensor would need to be combined with a watch, as people are already accustomed to wearing a watch and do not want to wear multiple things:

I don’t think I’d like things on my wrist—my wrist is my watch...it depends if that could all be one thing, that wouldn’t be too bad, but I don’t think I would have two things on my wrist, or one on each wrist. I don’t think I’d have that.

032TB

Of the 33 participants, The design of the sensor was also a common influencing factor, especially among the older group, as described by 8 (24%; younger women, n=2, 25%; younger men, n=1, 13%; older woman, n=3, 37%; and older men, n=2, 25%) participants:

I’d go for the watch because not all trousers, or skirts, have pockets, erm, and a ring—I’m very fussy with the rings I wear. But I very much like the watch. I think that looks really nice actually

025JD

One of the participants stated that one of the main influencing factors for them was curiosity (older man, 1/33, 3%):

it would be interesting, I don’t know if it would, you know, be useful. Or whether I would get, personally, anything out of it. But it would certainly allow me, if I wanted to take a scientific interest, to be able to analyse it. Just out of curiosity really.

Among the women of the 33 participants, health status was the main factor that would make them consider using a wearable system in the future, as described by 3 (9%; younger women, n=2, 67%, and older woman, n=1, 33%) participants:

I suppose if you get to a point or stage where you require that then I would want it but we don’t require it, and we hope we won’t

016IA

##### Advantages of Wearable Monitoring Systems

Of the 33 participants, one of the main advantages of the community-dwelling older adults related to wearable systems is the ability to monitor health, either their own or that of someone else, as described by 3 (9%; younger woman, n=1, 33%; younger man, n=1, 33%; and older woman, n=1, 33%) participants:

Yeah, I think if its monitoring heart rate and stuff like that—the movement—that basically covers what these things will do. This firstly then these secondary. A combination of both, but predominantly the [wearable] one. That definitely gives you more details of your personal health

023DK

Of the 33 participants, motivation was another key advantage identified by both groups, especially in relation to exercise, as described by 10 (30%; younger women, n=1, 10%; younger men, n=4, 40%; older women, n=3, 30%; and older men, n=2, 20%) participants:

I’ve set myself a target of going on a little walk every day to build up my fitness again, so I’m just trying to—just out of curiosity, seeing how far I’m going every day

007JR

Of the 33 participants, the unintrusive nature of the sensors and the fact that they can be hidden was a major advantage for several groups, as described by 10 (30%; younger men, n=2, 20%; older women, n=5, 50%; and older men, n=3, 30%):

I mean, that could be hidden. You’ve got it on and it’s hidden up a sleeve, you’re not going to be able to see it...the strap around the waist could be hidden. It could be easily hidden underneath clothing and then, you know, if you’re wearing a jacket or something as well it’s not going to be seen, and people wouldn’t ask

023DK

##### Disadvantages of Wearable Monitoring Systems

Older women noted that the potential need to charge the system could be a disadvantage of wearable monitoring systems (older women, 2/33, 6%):

oh yes, charging, that’s the thing

005PC

Of the 33 participants, both groups suggested that comfort may be a barrier, as described by 2 (6%; younger man, n=1, 50%, and older woman, n=1, 50%) participants:

I don’t think I would find it a problem unless it affected my sleep, you know, if it was uncomfortable and woke me up.

027BB

Younger men suggested that the cost of the system would discourage them from using wearable sensors (younger men, 2/33, 6%):

I mean I’ve always been quite, sort of, interested in the Fitbits and that sort of thing—the physical activity monitors and that but never...I’ve never wanted to spend that much money”

010BC

As these systems are wearable, of the 33 participants, the possibility of losing, forgetting, or damaging the sensor was the most stated disadvantage by 17 (52%; younger women, n=6, 35%; younger men, n=5, 29%; older women, n=2, 12%; and older men, n=4, 24%) participants:

I’d wear one on a belt but I know what I’m like for losing things, and if I had that one in my pocket I’d probably lose it...or put it in the washing machine

007JR

Of the 33 participants, the practicality of everyday use was another commonly stated disadvantage by 11 (33%; younger women, n=3, 27%; younger men, n=4, 36%; older women, n=3, 27%; and older man, n=1, 9%) participants, especially concerning ring and pocket sensors:

I think the ring can pose a problem, particularly of you are doing work, you can actually catch the ring in something and harm your finger. And erm, I mean, you know, I wear a wedding ring and another ring and I take those off if I am going to do some work for safety reasons. So I think you would be taking that off I would imagine, and perhaps forgetting to put it back on again

019GW

Younger men stated that the reaction of other people was a key disadvantage, especially in relation to appearing vulnerable (younger men, 2/33, 6%).

Because people will be saying “well what’s that” you know what I mean? It’ll be people saying “why you wearing this” and you’ll have to start making excuses. You don’t want to come across as being vulnerable

001WB

##### Overview of Environmental Sensors

There was little existing knowledge of environmental monitoring systems: 29% (5/17) of the younger group and 6% (1/16) of the older group were aware of at least one type (Figure 3). One of the participants explained that they had worked with the floor and chair sensors (younger man, 1/33, 3%):

at a home for people with dementia so we had the mats and the chair sensor to basically monitor when they were getting out of bed. Put a foot on the floor, the beeper would go off and we would go and see if they’re okay. Y’know especially at night times. Some people who are at risk of falling, we had the chair exit pads but not all this other stuff

001WB

One of the participants recalled seeing something similar to a passive infrared monitoring system on a television program (younger man, 1/33, 3%):

I’ve seen some similar, somewhere I’ve seen similar to the PIR setup in a room for motion sensor, just to check when people are actually moving. I can’t remember where—it might have been something like “Tomorrow’s World” [British science and technology TV programme which ran until 2003] or something like that. I saw it years ago

023DK

Of the 33 participants, experience with family or friends was mentioned by 2 (6%; younger woman, n=1, 50%, and older woman, n=1, 50%) participants:

I’ve heard about the anti-wandering ones because my friend’s mother-in-law had one of those [laughs]—and the bed

020PP

The younger group was more accepting of environmental sensors overall, with just 6% (1/17) saying they would not consider their use compared with 19% (3/16) of the older group. However, the younger group was more likely to consider using environmental sensors in the future (13/17, 76%) than immediately (3/17, 18%) compared with the older group (6/16, 38%, would consider using it in the future, and 7/16, 44%, would use it immediately).

**Figure 5:**
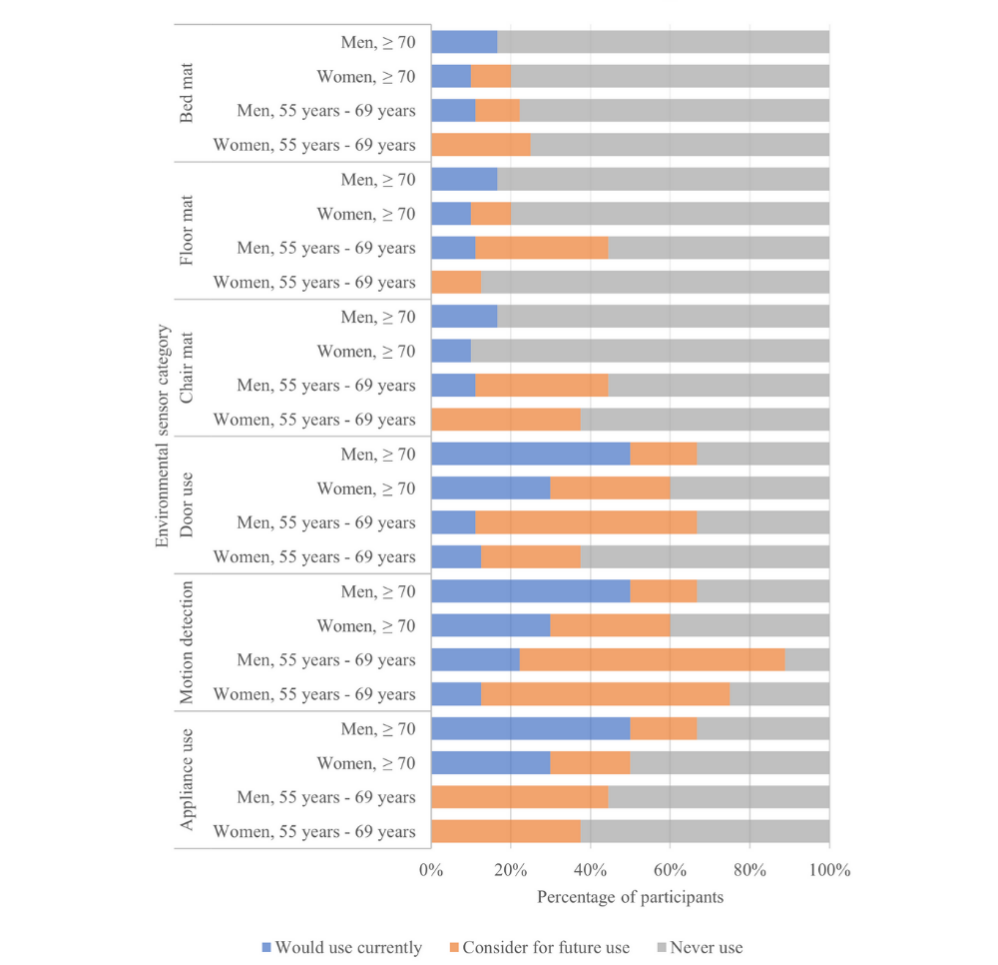
Acceptance of environmental sensors among community-dwelling older adults.

The acceptance of each specific environmental technology type is summarized in Figure 5. Motion sensors were the most accepted form of technology in the younger group, whereas motion and door sensors were most accepted equally by the older group. The pressure sensors were least accepted by both groups; however, several of the younger group participants would consider the chair mat in the future.

##### Factors Influencing Potential Acceptance of Environmental Monitoring Systems

Of the 33 participants, the main factor influencing community-dwelling older adults’ acceptance of environmental monitoring systems in both groups was the perception of usefulness, with many stating that they did not see why they would be useful, as described by 12 (36%; younger women, n=3, 25%; younger men, n=3, 25%; older women, n=3, 25%; and older men, n=3, 25%) participants:

I think we would know what we are doing. I don’t think we’d need data to tell us what we were doing...personally, I don’t think it would help, I don’t think it would make any difference to us to see it written down in the data round the house

028PG

Of the 33 participants, health status was identified by 9 (27%; younger woman, n=1, 11%; younger men, n=3, 33%; older women, n=4, 44%; and older man, n=1, 11%) participants:

if you’re not particularly able then I would think the chair monitor—you know, you do not want people sitting day-in, day-out and not moving. If I was unfortunate enough to be struck down with something like dementia then the one on the door would be essential—on the front door would be essential

025JD

Of the 33 participants, media influence was both a positive and negative factor for 3 (9%; younger woman, n=1, 33%; older woman, n=1, 33%; and older man, n=1, 33%) participants:

yeah, I’d be like James Bond wouldn’t I with sensors all over the place! Yeah that would be lovely. Yeah, excellent.

014PC

I don’t fancy them, it’s a bit “big brother is watching you”

021GD

##### Advantages of Environmental Monitoring Systems

Of the 33 participants, reassurance not only for the individual but also for those caring for an older relative was the most stated advantage of having an environmental monitoring system in both groups, especially for the younger group, as described by 11 (33%; younger women, n=5, 45%; younger men, n=3, 27%; and older women, n=3, 27%) participants:

You never know what’s around the corner, then I would actually feel reassured with all this stuff at the top

001WB

I think it’s—for people that have been through that or whatever sort of illnesses you’ve had with your elderly parents or whoever, this would probably be quite reassuring...I would have loved something like that for my mum. It would have been brilliant.

012SH

One of the participants stated that having an environmental sensor may allow certain conditions to be diagnosed earlier than otherwise (younger women, 1/33, 3%):

my mum had dementia and I was constantly getting called to her flat, something like this before she got to that stage where it got messy, these things could probably have diagnosed her earlier

012SH

Of the 33 participants, mostly the women in both the older and younger groups stated that a key advantage would be identifying sedentary behavior, either in themselves or others, as described by 7 (21%; younger women, n=2, 29%; younger man, n=1, 14%; and older women, n=4, 57%) participants:

if it got to the stage where I did need that, I mean, bearing in mind that there’s always the threat of DVT if you spend too long sitting down. I mean, if you have somebody who’s had a stroke or something and they aren’t moving around much, I think that would be very informative.

025JD

Of the 33 participants, safety was also mentioned by 3 (9%; younger woman, n=1, 33%; younger man, n=1, 33%; and older woman, n=1, 33%) participants in both the older and younger groups, relating to personal safety for 2 (6%; younger woman, n=1, 50%, and older woman, n=1, 50%) participants and the potential for home security for 1 (3%; younger man) participant:

I suppose if you had someone with dementia, it would tell you if they’d been out, or gone out when they shouldn’t.

025JD

if there’s only 1 person in the house, and then all of a sudden there’s three or four things moving about—there’s somebody in a bedroom, someone in the living room, and then you think “this person lives on their own,” but there’s movement in two or three different places at one time—a quick phone call or something like that...Rather than find out later that they’ve been burgled

023DK

Of the 33 participants, the unintrusive nature of the sensors was a key advantage, especially within the older group when compared with wearable sensors, as described by 4 (12%; younger woman, n=1, 25%; older women, n=2, 50%; and older man, n=1, 25%) participants:

Obviously the ones that are installed on your ceiling or on your wall are not obtrusive at all, whereas you’ve got to remember to wear the other thing and of course you would realise that you have always got it with you

019GW

##### Disadvantages of Environmental Monitoring Systems

One of the participants identified the potential cost of the system as a disadvantage (younger woman, 1/33, 3%):

Expense wise, a little one would be cheaper than putting something in every room. That’s—you know, finance is another issue

024SK

Of the 33 participants, the issue of coping with habitual behavior, such as closing doors, was identified by 4 (12%; younger woman, n=1, 25%; younger man, n=1, 25%; and older women, n=2, 50%) participants, especially in relation to door sensors:

door one, um, apart from the front doors, wouldn’t work too much for me because I tend to not close doors anyway

020PP

Of the 33 participants, 2 (6%; younger man, n=1, 50%, and older woman, n=1, 50%) participants stated that the environmental sensors posed a risk of damage to the house:

it’s worth noting, because one question would be if it does mark, then people would say “well then, I’ve got to redecorate”

027BB

Of the 33 participants, concerns over privacy issues were identified by most groups, as described by 5 (15%; younger women, n=3, 60%; older woman, n=1, 20%; and older men, n=1, 50%) participants:

Someone is always listening to you, someone is always looking at you, that the only thing. There isn’t much privacy there, is it?

018CW

One of the participants stated that they considered the potential for reduced human contact within health care to be a disadvantage (older woman, 1/33, 3%):

the only slight misgiving I have on that is, um, that if—that they could end up, sort of, replacing the one-to-one...So these would keep you safe, say, these would alert somebody to a situation perhaps, or give them information but they couldn’t replace the, sort of, the human contact element

021GD

#### Future Development Considerations of Monitoring Systems in General

Following the discussion of the various currently available monitoring technologies, some participants highlighted ways in which they could be developed in the future. Of the 33 participants, the main outcome from the younger group was that any future development should be based on a specific need, as described by 2 (6%; younger woman, n=1, 50%, and younger men, n=1, 50%) participants:

yep, if something’s got a use and it makes life easier then I’m all for it, but if someone has invented some technology and then tries to find a use for it, I don’t think that is a great improvement...If there is a need, get the technology to deal with it rather than develop technology and then find a use for it...We should get the machine to do whatever it wants to do properly and reliably rather than find out what else you can make it do—unless it is of some use.

017SC

One of the participants highlighted the need for more education relating to these types of technology (younger man, 1/33, 3%):

Yeah so I guess it’s the educational side of it, yes. Teaching them about it—making sure the IT works for them rather than it’s just there

003MC

One of the participants suggested that being able to combine many measures into a single sensor may be beneficial rather than having multiple sensors (older woman, 1/33, 3%):

it would be better to have one that would just do the lot rather than one that just picks one thing out, really...One that could combine the whole lot would be better.

006BR

## Discussion

### Principal Findings

#### Overview

Although there have been studies on older adults’ perceptions of assistive technology [11] and fall monitors [13], little is known about their perceptions of ADL monitoring technology. A recent review of ADL monitoring technology found that there is a need for a clear consensus on which ADL are important to monitor and what types of technology older adults are most likely to use to enhance the effectiveness of current ADL monitoring systems [9]. The sample size used in this study, as well as the inclusion of both younger and older groups, makes this one of the most comprehensive studies of older adults’ perceptions of ADL monitoring technology to date. PEIs made it possible to clarify the perceptions of specific types of technologies; however, the images used in PEIs were not exhaustive examples of the types of monitoring technology available. It should also be noted that all participants were from the United Kingdom and were already comfortable using technology, which means that the following conclusions cannot necessarily be applied globally. Differences in aspects such as education level and access to technology may also alter the results, although this was not a specific consideration of this study.

#### Relationship Between ADL Importance and Monitoring Technology

There is currently a strong link between the ADL considered the most important by the community-dwelling older adults and the ADL most frequently identified by current monitoring systems, and feeding and personal hygiene activities are both the most common activities supported by monitoring systems [9] and are considered the most important activities by community-dwelling older adults. In this study, these are also the activities that community-dwelling older adults suggested they would be the least likely to accept or ask for help with, particularly those relating to personal hygiene, such as washing and toileting, which were closely associated with feelings of pride and dignity.

One of the main challenges in developing ADL monitoring technology is identifying the activities that are important for monitoring [9]. Although hygiene-related activities are considered important, many older adults express concern about them being directly monitored [20], and they often require several sensors that focus only on these activities [9]. The activities considered important by community-dwelling older adults were different between the younger and older groups, with those aged ≥70 years placing more importance on stair use than their younger counterparts; however, this is not commonly detected by ADL monitoring systems. Instead, it often features in sensors specifically designed to monitor falls; however, the requirement for several different, highly specialized systems may be alienating some community-dwelling older adults. It was highlighted that most community-dwelling older adults were inclined toward fewer sensors, suggesting that a simple sensor capable of monitoring several activities would be preferable, which highlights the potential need for collaboration between those developing fall technology and ADL monitoring technology.

It was also noted that some activities have a large influence on others, namely, mobility and standing from sitting in a chair, which are both required to perform almost any other ADL. This suggests that it might be less important to directly monitor specific activities; instead, the focus should be on movements that are considered the most influential. For example, squatting plays a role in sitting, toilet use, and potentially other activities. Further investigation is required to identify the link between functional movement and specific ADL. Physical ability was a theme shared by both ADL performance and technology acceptance, highlighting that it is a very influential aspect in the acceptance of monitoring technology by the community-dwelling older adults.

#### Link Between Existing Knowledge of Technology and Acceptance of ADL Monitoring Technology

Our results demonstrated that there were different levels of existing knowledge related to monitoring technology, with most people being aware of wearable sensors and very few being aware of environmental sensors. One of the key reasons for this is the presence of similar technology in general use; for example, many noted that wearable sensors resemble smart watches such as Fitbit. It is evident that there is a link between existing knowledge and acceptance, as wearable sensors had the highest previous knowledge and acceptance, whereas environmental sensors had the lowest score in both categories (Figure 3). This contrasts with existing studies, which have suggested that wearable sensors are often the least accepted [21-23], whereas environmental motion sensors have been the most accepted [24]. It is notable that the younger group had more experience with environmental sensors than the older group, which many of them stated was because of having older relatives who had used the technology. The younger group was also more accepting of environmental sensors as they could relate their potential use to their relatives and wanted to have sensors that could help reassure their future carers or potentially help them diagnose health concerns earlier. On the basis of this, it may be that as monitoring technology becomes more commonly used, it will also become more accepted.

The high acceptance of monitoring technology in this study may be due, in part, to the sample group, who were all accepting of technology in general and comfortable using it, hence their willingness to use video calling software to participate in the interview. It should be noted that this may not be representative of the entire older adult community; for example, Verloo et al [11] suggest that older adults have limited interest in technology, and therefore, generalizations are difficult. However, this study was also conducted during the UK COVID-19 lockdown restrictions, during which technology became more prominent in many people’s lives as a means of maintaining social connections with family and friends. Alongside this, some participants also noted that because of limited social interaction, people might be less aware of medical emergencies or emerging health concerns than they may have been previously and therefore were more appreciative of having technology in the home to monitor things such as sedentary behavior than they may have been in the past.

#### Common Themes

Some common themes emerged across both technology types, namely, relating to health status as an influencing factor and cost as a disadvantage. Health status highlights the potential of these sensors to be used to support health care, which is one of the main objectives for their development [9,12,25,26]; however, many of the participants highlighted that they would not use these sensors until they needed them, which goes against the idea of using them to detect when this point of need may be occurring. Owing to their ability to monitor continuously, which human health care workers cannot [20], it may be beneficial to focus future work on highlighting how this may be beneficial to older adults. Particular emphasis should be placed on older adults at most risk of becoming frail or developing certain health conditions such as dementia. However, the fact that cost was highlighted as the main disadvantage across all technology types shows the prominence of this issue. This sentiment is echoed by several other studies that have been conducted over several years, demonstrating that this is a key issue for developers to overcome in future development, finding a technology that is both beneficial and cost-effective [3,11].

A key disadvantage was the potential for reduced human contact, particularly among older women. This may be linked to a higher incidence of loneliness among this population because of the unequal distribution of risk factors such as the death of a partner among men and women [27] and the subsequent need to maintain social relationships. Although these technologies are often developed to assist health care and allow older adults to live at home for as long as possible [9], the development of future systems should be careful not to completely replace human care with technological assistance. Human interaction can provide emotional connections that even the smartest technology cannot replicate. These emotional connections cannot be underestimated in the care of older adults, as they are known to be closely linked with other factors such as depressive symptoms and subsequent reductions in physical activity and overall health. However, it is possible to use sensors to reduce the workload for human carers by automatizing some tasks; therefore, the carers can be more available to provide more human interaction to older users.

Socializing and communication were considered among the most important ADL by community-dwelling older adults and are commonly identified by monitoring technology [9], despite not appearing on many traditional ADL scales [28]. Social interaction is becoming an increasingly prevalent aspect of health care because of the growing adoption of a biopsychosocial approach [29]; therefore, this study indicates that its presence in monitoring technology should continue. Although not included in this study, socially assistive robots (SARs) may represent the best opportunity for developing this, as they have already been shown to have benefits for socialization [13,30]. In addition to monitoring socializing activities, SARs may play an active role in supporting the community-dwelling older adults through conversation or facilitating communication between people. It should be noted that this study was conducted during the national lockdowns in response to the COVID-19 pandemic, which several participants noted had made them more aware of potential isolation from friends and relatives. Future work should include SARs and explore their potential usefulness in the monitoring of ADL performance, as well as their role in supporting community-dwelling older adults to continue living independently in the community.

### Conclusions

Overall, technological monitoring systems are perceived as acceptable methods of monitoring ADLs. However, developers and carers must be aware that individuals may express differences in their willingness to engage with certain types of technology depending on their age and circumstances. In addition to the increase in population aging, there will be an increase in older adults with interest in technology, which may reduce some of the existing barriers [11]; however, technical developers should continue to ensure that technology is created for a specific purpose that can be clearly conveyed to community-dwelling older adults who may not have much technological experience. Community-dwelling older adults highlighted the need for systems to be combined and simple; they do not want multiple sensors as these can create a technology overkill. In the future, technical developers should consider this and note that as technology becomes more widespread, it will become more accepted.

## Appendix

Multimedia Appendix 1 Interview guide used for web-based interviews.

Multimedia Appendix 2 Complete participant quotes from web-based interviews.

## Abbreviations

ADL: activities of daily living
GARS: Groningen Activity Restriction Scale
iADL: instrumental activities of daily living
PEI: photo-elicitation interview
SAR: socially assistive robot
