# Supplementary material for: Perceptions of In-home Monitoring Technology for Activities of Daily Living: Semistructured Interview Study With Community-Dwelling Older Adults
Source: JMIR Aging. 2022 May 5;5(2):e33714. doi: 10.2196/33714 (PMC9121226; doi:10.2196/33714)

**Section 1: ADLs, wearable/environmental tech & current measures**

Section aims:

- To understand what older adults view as important, fundamental activities of everyday life and what difficulties they face with them (e.g. physical function/cognitive function)
- To understand what technology older adults are familiar with, in terms of measuring/monitoring ADLs (wearable technology / environmental sensors)
- To understand older adults’ concerns surrounding current technology in the home (environmental sensors, wearable technology)

Questions:

1. What is your understanding of “activities of daily living”?
   1. What activities do you consider fundamental to your daily life?
   2. What aspects of your life make these more difficult / what do you expect the difficulties to be as you age?
      1. Physical difficulties such as balance, weakness, grip strength
      2. Cognitive difficulties such as memory loss/forgetfulness

**Give list from GARS scale and rate them (this scale includes ADL & iADL)**

1. What is your understanding of “wearable technology” in terms of human activity monitoring?

**Show examples of IMU sensors and briefly explain their function (screen share / email in advance)**

- 1. What do you like/dislike about the technology shown here?
  2. What do you think the benefits of using this technology to monitor activities of daily living might be?
  3. What concerns do you have with the use of these types of technology?

1. What is your understanding of “environmental sensors” in terms of human activity monitoring?

**Show examples of environmental sensors and briefly explain their function (screen share / email in advance)**

- 1. What do you like/dislike about the technology shown here?
  2. What do you think the benefits of using this technology to monitor activities of daily living might be?
  3. What concerns do you have with the use of these types of technology?

1. Would you prefer using wearable technology, environmental sensors, a combination of both or neither?
   1. Why?

**Wearable Sensors**


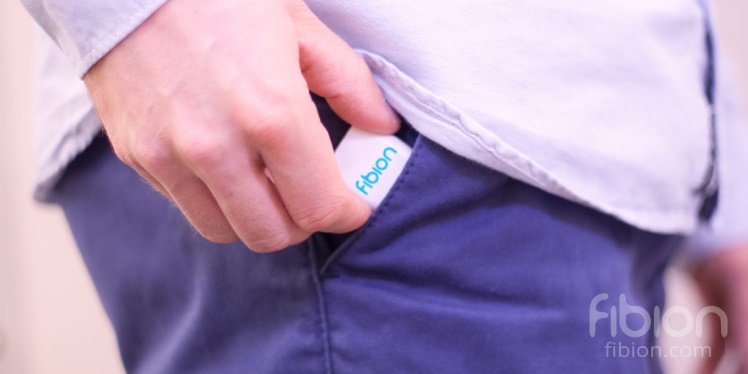

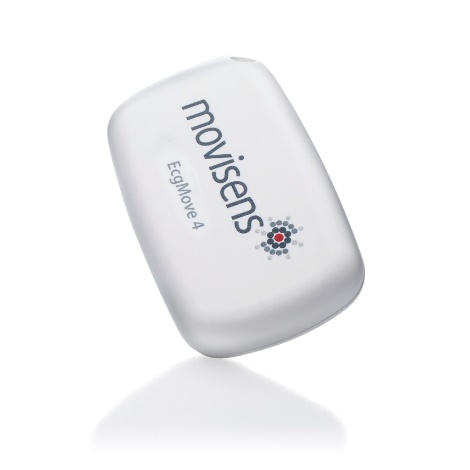


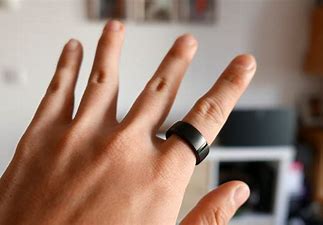

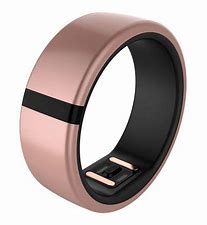

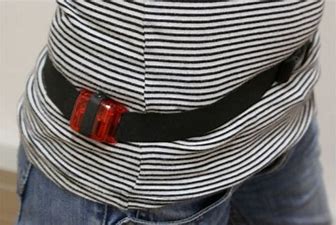

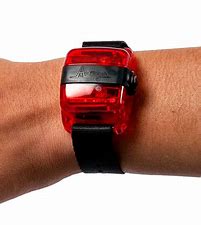

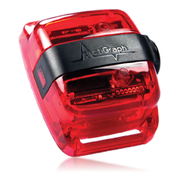


**Environmental Sensors**


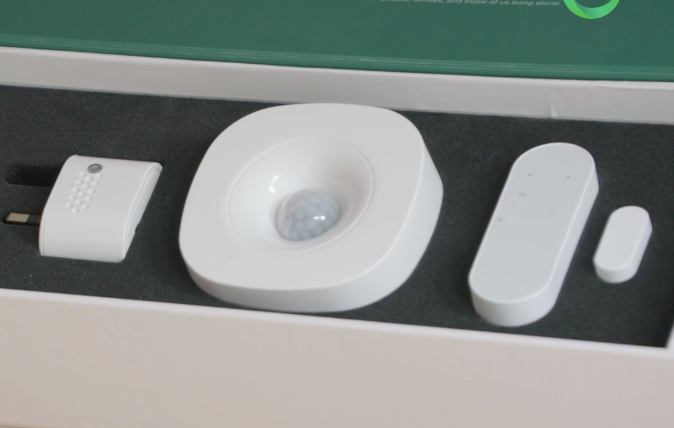


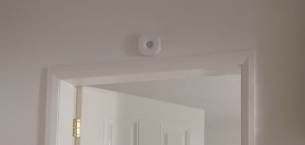

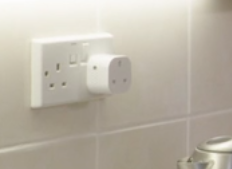


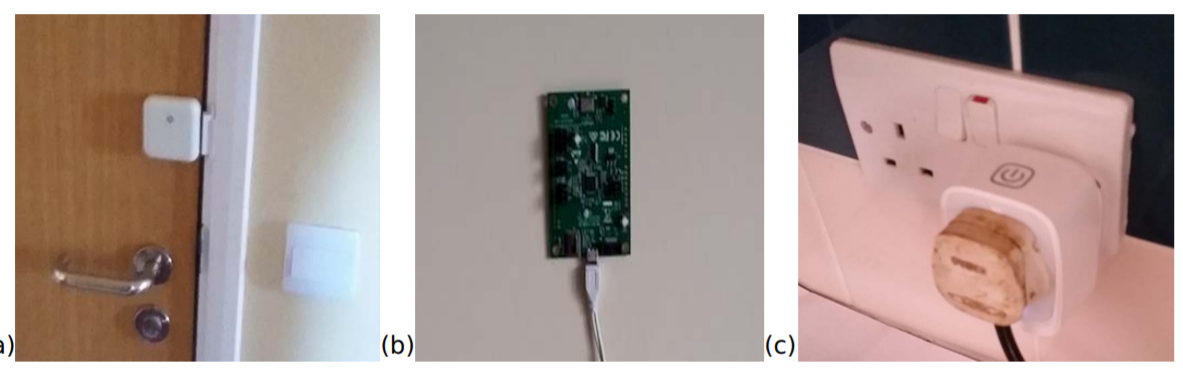


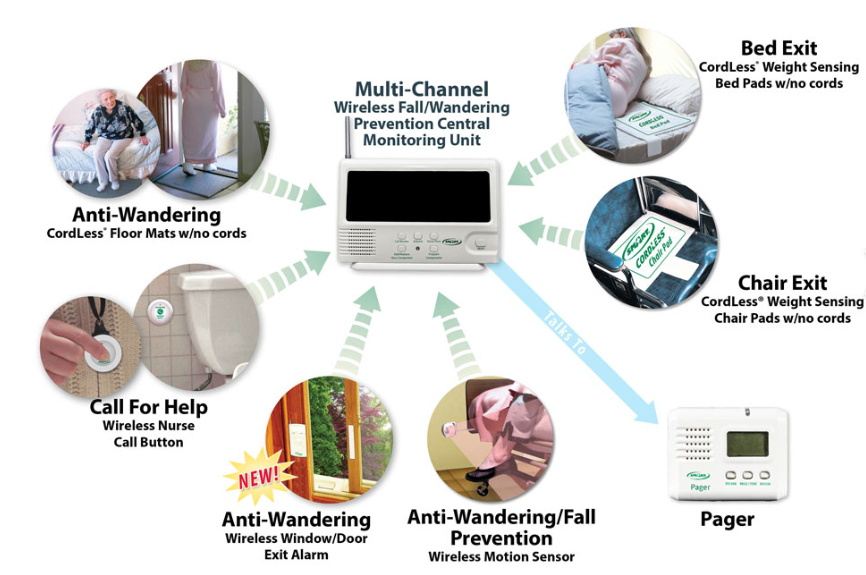


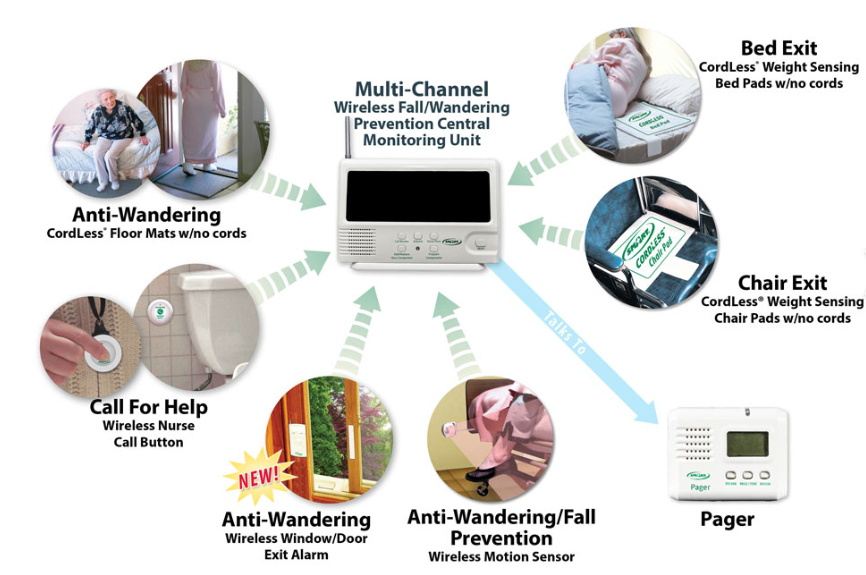

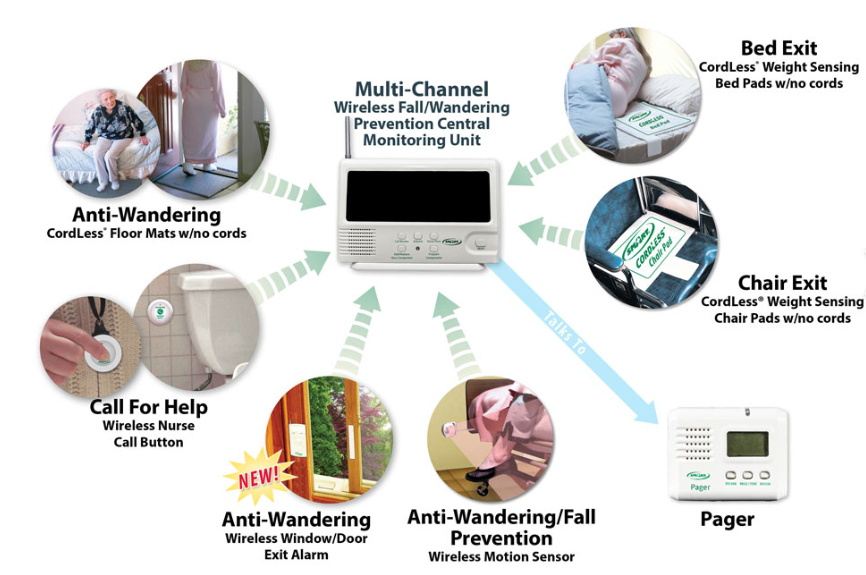

Supplement: Multimedia Appendix 1 [file aging_v5i2e33714_app1.docx]
